# Content validity of the NECPAL CCOMS-ICO© in Spanish to identify palliative needs in children and adolescents with Cancer*

**DOI:** 10.17533/udea.iee.v40n1e06

**Published:** 2022-03-29

**Authors:** Julián Alberto López Alba, Diana Marcela Jaramillo García, Nadia Carolina Reina Gamba

**Affiliations:** 1 Nurse, Masters in Palliative Care Nursing. Universidad El Bosque, Bogotá (Colombia). Email: julopeza@unbosque.edu.co, julianlopez247@gmail.com. Corresponding author. Universidad El Bosque Universidad El Bosque Bogotá Colombia julopeza@unbosque.edu.co julianlopez247@gmail.com; 2 Nurse, Masters in Palliative Care Nursing. Universidad El Bosque, Bogotá (Colombia). E-mail: djaramillog@unbosque.edu.co, dmjaga.3@gmail.com. Universidad El Bosque Universidad El Bosque Bogotá Colombia djaramillog@unbosque.edu.co dmjaga.3@gmail.com; 3 Nurse. PhD. Professor, Universidad Nacional de Colombia, Bogotá (Colombia). Email: ncreinag@unal.edu.co Universidad Nacional de Colombia Universidad Nacional de Colombia Bogotá Colombia ncreinag@unal.edu.co

**Keywords:** neoplasms, child, palliative care, needs assessment, surveys and questionnaires, validation study., neoplasia, niño, cuidados paliativos, evaluación de necesidades, encuestas y cuestionarios, estudio de validación., neoplasia, criança, cuidados paliativos, determinação de necessidades de cuidados de saúde, inquéritos e questionários, estudo de validação.

## Abstract

**Objective.:**

To perform content validation of the NECPAL CCOMS-ICO© instrument to identify palliative needs in Colombian children and adolescents from 8 to 17 years of age with cancer.

**Methods.:**

Psychometric study, which used the Lawshe model, modified by Tristan, to perform content validity of the NECPAL CCOMS-ICO© instrument through expert consensus.

**Results.:**

The Surprise Question (SQ) *Would it surprise you if this patient died within the next year?* and the parameters *Demand: Has there been any implicit or explicit expression of limitation of therapeutic effort or demand for palliative care from the patient, family, or team members?*, *Need: identified by professional members of the team*, *Checklist symptoms (ESAS) ≥ 2 persistent or refractory symptoms, Emotional Distress Detection (EDD) > 9*, *Social and family assessment*”, *Oncological disease (advanced metastatic or locoregional cancer)*, *Oncological disease (in progression (in solid tumors)* and *Oncological disease (Persistent symptoms poorly controlled or refractory despite optimizing specific treatment)*, were considered valid by the experts to identify palliative needs in Colombian children and adolescents aged 8 to 17 years with cancer according to the Lawshe criteria, as modified by Tristán. Parameters specifically related to the oncological disease belonging to the dimension *Indicators of severity/progression of the disease* showed the highest CVR’ per parameter, with indices of agreement between 0.714 and 0.857.

**Conclusion.:**

The content of the NECPAL CCOMS-ICO © 3.1 instrument is valid to identify palliative needs in Colombian children and adolescents from 8 to 17 years of age with cancer with a CVI of 0.68.

## Resumen

**Objetivo.:**

Realizar validación de contenido del instrumento NECPAL CCOMS-ICO© para identificar necesidades paliativas en niños y adolescentes colombianos, entre 8 y 17 años, con cáncer.

**Métodos.:**

Estudio psicométrico que utilizó el modelo de Lawshe modificado por Tristán para evaluar la validez de contenido del instrumento NECPAL CCOMS-ICO© a partir del consenso de expertos.

**Resultados.:**

La Pregunta Sorpresa *¿Le sorprendería que este paciente muriese a lo largo del próximo año?* y los parámetros de *Demanda: ¿Ha habido alguna expresión implícita o explícita de limitación de esfuerzo terapéutico o demanda de atención paliativa de paciente, familia, o miembros del equipo?*, *Necesidad: identificada por profesionales miembros del equipo*, *Checklist síntomas ≥ 2 síntomas persistentes o refractarios*, *Detección de Malestar Emocional > 9, Valoración social y familiar*, *Enfermedad oncológica (Cáncer metastásico o locoregional avanzado)*, *Enfermedad oncológica (en progresión (en tumores sólidos)* y *Enfermedad oncológica (Síntomas persistentes mal controlados o refractarios a pesar de optimizar el tratamiento específico)*, se consideraron válidos por los expertos para la identificación de necesidades paliativas en niños y adolescentes colombianos, entre 8 y 17 años, con cáncer, de acuerdo con los criterios de Lawshe modificados por Tristán. Los parámetros relacionados específicamente con la enfermedad oncológica pertenecientes a la dimensión *Indicadores específicos de severidad y progresión de la enfermedad* presentaron los mayores valores de la Razón de Validez de Contenido por parámetro que oscilaron entre 0.714 y 0.857.

**Conclusión.:**

La validez de contenido del instrumento en español NECPAL CCOMS-ICO© mostró ser apropiada para identificar las necesidades paliativas en niños y adolescentes colombianos, entre 8 y 17 años, con cáncer.

**Objetivo.:**

Realizar validação de conteúdo do instrumento NECPAL CCOMS-ICO© para identificar necessidades paliativas em crianças e adolescentes colombianos de 8 a 17 anos com câncer.

**Métodos.:**

Estudo psicométrico que utilizou o modelo de Lawshe modificado por Tristán para avaliar a validez de conteúdo do instrumento NECPAL CCOMS-ICO© através do consenso de especialistas.

**Resultados.:**

A Pergunta Surpresa. *Lhe surpreenderia que este paciente morresse ao longo do próximo ano?* e os parâmetros de *Demanda: Tem tido alguma expressão implícita ou explícita de limitação de esforço terapêutico ou demanda de atenção paliativa de paciente, família, ou membros da equipe?*, *Necessidade: identificada por profissionais membros da equipe*, *Checklist sintomas ≥ 2 sintomas persistentes ou refratários*, *Detecção de Mal-estar Emocional > 9, Valoração social e familiar*, *Doença oncológica (Câncer metastático ou locorregional avançado)*, *Doença oncológica (em progresso (em tumores sólidos)* e *Doença oncológica (Sintomas persistentes mal controlados ou refratários apesar de otimizar o tratamento específico)*, foram considerados válidos pelos especialistas para a identificação de necessidades paliativas em crianças e adolescentes colombianos de 8 a 17 anos com câncer, de acordo com os critérios de Lawshe modificados por Tristán. Os parâmetros relacionados especificamente com a doença oncológica pertencentes à dimensão *Indicadores específicos de severidade e progressão da doença* apresentaram os maiores valores da Razão de Validez de Conteúdo por parâmetro que oscilaram entre 0.714 e 0.857.

**Conclusão.:**

A validez de conteúdo do instrumento em espanhol NECPAL CCOMS-ICO© mostrou ser apropriada para identificar as necessidades paliativas nas crianças e adolescentes colombianos de 8 a 17 anos com câncer.

## Introduction

According with data provided by the National Public Health Surveillance System in Colombia, the mortality rate due to cancer is estimated at two for every 100,000 minors under 18 years of age, which includes deaths due to acute pediatric leukemia.(1) When patients are diagnosed in terminal phase, health professionals must determine and assess the needs caused by the late effects that cancer brings as a catastrophic disease in the physical, mental, social, and psychological spheres.(2)

With respect to the information offered by the Work Group Palliative Care for Children of the European Association for Palliative Care, the estimated European prevalence rate for children and young people who may require palliative care is of 10 to 16 for every 10,000 inhabitants between 0 and 19 years of age (15 for every 10,000 if neonatal deaths are excluded), of which approximately 30% of these patients suffer from cancer, which would mean that, in a population of 250,000 people in which there are about 50,000 children, in one year there is a probability that eight children will die from life-limiting diseases, 37.5% as a consequence of cancer; 60 to 80 would suffer a life-limiting disease; 30 to 40 of them would need specialized palliative care, that is 50%.(3)

According to figures from the Colombian Ministry on Health and Social Protection, the Department of Epidemiology and Demographics and the National Cancer Observatory (ONC, for the term in Spanish) in Colombia, in the 2014 report, the age group with the highest mortality rate due to childhood cancer between 2005 and 2011 corresponds to minors between 10 and 18 years of age.(4) The aforementioned coincides with the 2016 report by the Colombian Fund for High-Cost Diseases on the distribution of childhood cancer, according to age group and gender, which denotes that the most-affected age group was comprised by those between 10 and 14 years of age.(5) The departments that were most affected were: Bogotá D.C., followed by Meta, Caldas, Antioquia, Valle del Cauca, Santander, Huila, Quindío, and Risaralda with a prevalence range from 331 to 543 per million inhabitants under 18 years of age.(5)

Given this scenario, one of the main challenges health professionals must face is to identify these palliative needs in people with life-threatening diseases, such as cancer. Internationally, instruments have been validated, like the IDC-Pal(6) and the PCP(7), to determine the amenable complexities and the phase in palliative care in which a patient is and others, like the RADPAC,(8) SPICT-ES™,(9) and NECPAL CCOMS-ICO©(10) to identify palliative needs; nevertheless, although the last two are validated in Spanish, none of those mentioned has been used in children and adolescents with cancer, unlike the PaPas Scale,(11) which was validated specifically in this type of population - but in English - and has five domains (Life expectancy, Expected outcome of disease-directed treatment, Performance status, Symptom burden and Problems and Preferences of the patient, family, or health professional). The aim of this study was to validate the contents of the NECPAL CCOMS-ICO© instrument to identify palliative needs in Colombian children and adolescents from 8 to 17 years of age with cancer.

## Methods

This was a psychometric study, using the Lawshe model(12) modified by Tristán,(13) to determine the content validity of the NECPAL CCOMS-ICO© instrument - adapting it to identify palliative needs in Colombian children and adolescents from 8 to 17 years of age with cancer - through the assessment by seven Colombian professional medical experts of which one is a specialist in anesthesiology and interventionism in pain and palliative care with a master's degree in pain management, one is a specialist in pediatrics and pediatric oncology, three are specialists in pediatrics and pediatric palliative care, one is a specialist in family medicine and pediatric palliative care, and one is pediatrist specialist in pediatric haemato-oncology and doctor in medicine. All the experts have between 5 and 26 years of care experience in their area of expertise, as well as teaching experience between 5 and 23 years and research experience between 4 and 24 years.

The NECPAL CCOMS-ICO© instrument, developed by Gómez *et al.,*(10) was created from a Spanish adaptation, the Prognostic Indicator Guidance (PIG) scales(14) and the Supportive & Palliative Care Indicators Tool (SPICT)(15) in a joint initiative by the QUALY Observatory (WHO Collaborating Center for Public Programs on Palliative Care (CCOMS, for the term in Spanish) and the Catalan Institute of Oncology (ICO). The 3.1 version of the NECPAL CCOMS-ICO©, which dates to 2017,(16) was used by the authors in this study to conduct the psychometric study. This instrument, validated in Spain, is useful to identify people with advanced complex chronic processes, who are specially affected, have palliative needs and are in social and health services. It is comprised of a surprise question (SQ) *Would you be surprised if this patient died within the next year*, nine dimensions and the following parameters, as shown in Table 1.

**Table 1:** Dimensions of the *NECPAL CCOMS-ICO©* instrument version *3.1* 2017

*Surprise question (SQ)*	*Would you be surprised if this patient died within the next year?*	*Would you be surprised if this patient died within the next year?*
Dimensions	Parameter	Parameter
“Demand” or “Need”	Demand: Has there been any implicit expression or limitation of therapeutic effort or demand for palliative care from the patient, family, or staff members?	Demand: Has there been any implicit expression or limitation of therapeutic effort or demand for palliative care from the patient, family, or staff members?
“Demand” or “Need”	Need: identified by professional staff members	Need: identified by professional staff members
Overall clinical progression indicators	Nutritional decline	Weight loss > 10%
- The last six months	Functional decline	• Karnofsky or Barthel deterioration >30%
- Not related to recent/reversible intercurrent process		• Loss of >2 ADLs
	Cognitive decline	Loss >5 minimental or >3 Pfeiffer
Severe dependence	Karnofsky <50 or Barthel <20	Anamnesis clinical data
Geriatric syndromes	• Falls	• Anamnesis clinical data
	• Pressure ulcers	• ≥ 2 geriatric syndromes (recurrent or persistent)
	• Dysphagia	
	• Delirium	
	• recurrent infections	
Persistent symptoms	Pain, weakness, anorexia, digestive…	•Symptoms checklist (ESAS) >2 persistent or refractory symptoms
Psychosocial aspects	Distress and/or Severe adjustment disorder	Emotional Discomfort Detection (EDD) >9
Psychosocial aspects	Severe social vulnerability	Family social assessment
Multi-morbidity	>2 advanced chronic diseases or conditions (from the list of specific indicators)	>2 advanced chronic diseases or conditions (from the list of specific indicators)
Use of resources	Assessment of the demand or intervention intensity	• >2 emergency or unplanned admissions 6 months
		• Increased demand or intervention intensity (home care, nursing interventions, *etc*.,)
Specific indicators of severity/ progression of the disease	Oncological disease	• Advanced locoregional or metastatic cancer
		• In progression (in solid tumors)
		• Poorly controlled or refractory persistent symptoms despite optimizing specific treatment.

The instrument is available in Spanish and its interpretation considers that only if the answer to the first question (surprise) is negative, the remaining parameters are completed with which the NECPAL is deemed positive when the answer to the surprise question (SQ) was “*no”* and one or more of the other parameters was positive. This instrument has been subjected to validation processes in adult population in Latin American countries, like Chile, where a cultural adaptation was carried out, along with content validity, piloting, application, and statistical analysis of the NECPAL-CCOMS-ICO 3.1©;(17) Argentina, where the instrument was used to identify people with advanced chronic diseases and needs for palliative care in the city of Buenos Aires;(18) and Colombia, where Moreno and Peláez(19) conducted face validation of the NECPAL - CCOMS© instrument in Bogotá D.C by using professional health experts to verify comprehension, precision, and clarity of the instrument in the health staff.

The dimension of *geriatric syndromes* was not kept in mind because it was not applicable to the target population in this study and only included specific severity or progression indicators related with oncological disease, so that, in addition to the surprise question (SQ), the remaining parameters shown in Table 1 were submitted to the content validation process by experts.

Personal or telephone contact was established with the experts. Three documents were delivered to those who accepted (letter of invitation to participate, characterization sheet by the expert and content validation instructions), which contained a validation grid for professionals to evaluate the surprise question (SQ) and the parameters, being able to only select one of the following options: *Essential, Useful but not essential,* and *Not necessary*, besides having a grid to conduct the observations the expert considered pertinent.

After receiving the documents from the experts, the researchers gathered to analyze the information provided by said experts and a Microsoft Excel database was constructed to determine the Content Validity Rate (CVR and CVR’) and the Content Validity Index (CVI) for each of the items and, finally, the Instrument’s Content Validity Index, through the equations described in the Lawshe model(12) modified by Tristán(13) for which the CVI cut-off point must be ≥ 0.58 to consider the question and the parameters valid (preferring the CVI over the Kappa index because it permits a reduced number of experts in areas as specific as pediatric palliative care).

## Results

The results show that the Surprise question (SQ) *Would you be surprised if this patient died within the next year* and the parameters *Demand: Has there been any implicit expression or limitation of therapeutic effort or demand for palliative care from the patient, family, or staff members*, *Oncological disease (Advanced locoregional or metastatic cancer), Oncological disease (in progression (in solid tumors),* and *Oncological disease (Poorly controlled or refractory persistent symptoms despite optimizing specific treatment),* exceed the cut-off point of 0.58, which is why they are considered acceptable according with criteria by Lawshe(12) modified by Tristán.(13) Moreover, the authors in this study have decided to include in this same category the parameters of *Need: identified by professional staff members, Symptoms checklist (ESAS) ≥ 2 persistent or refractory symptoms, EDD > 9, Family and social assessment* given that, although they all scored at 0.5714, their value is very close to the cut-off point.

Besides the Surprise question (SQ)*,* of the 16 instrument’s parameters subjected to the content validation process, eight were considered pertinent by the experts to identify palliative needs in children and adolescents with cancer and those related specifically with the oncological disease belonging to the dimension *specific disease severity/progression indicators* had the highest CVR’ per parameter, with indices of agreement among experts ranging between 0.714 and 0.857. The global CVI was 0.6825. The CVR for each of the items are shown in Table 2.

**Table 2:** Content validity index for the NECPAL CCOMS-ICO© 3.1 parameters

Dimension/ Parameter	Score	Score	Score	CVR	CVR'
Dimension/ Parameter	Essential	Useful	Not necessary	CVR	CVR'
Demand or need					
Demand	5	2	0	0.4286	0.7143
Need	4	3	0	0.1429	0.5714
Progression indicators					
Nutritional decline	0	6	1	-1.0000	<0.0001
Functional decline *	2	3	2	-0.4286	0.2857
Functional decline**	3	2	2	-0.1429	0.4286
Cognitive decline	0	2	5	-1.0000	<0.0001
Severe dependence	1	4	2	-0.7143	0.1429
Persistent symptoms	4	3	0	0.1429	0.5714
Psychosocial aspects					
Distress and/or Severe adjustment disorder	4	2	1	0.1429	0.5714
Severe social vulnerability	4	2	1	0.1429	0.5714
Multi-morbidity	1	3	3	-0.7143	0.1429
Use of resources					
>2 emergency or unplanned admissions (six months)	2	4	1	-0.4286	0.2857
Increased demand or intervention intensity	3	4	0	-0.1429	0.4286
Severity or progression indicators					
Advanced locoregional or metastatic cancer	5	1	1	0.4286	0.7143
In progression	6	1	0	0.7143	0.8571
Poorly controlled or refractory persistent symptoms	6	1	0	0.7143	0.8571
Surprise question: Would you be surprised if this patient died within the next year?	5	2	0	0.4286	0.7143

* Karnofsky or Barthel deterioration >30% ** Loss of >2 ADLs

Table 3 evidences that the CVI by expert is adequate because it exceeds the cut-off point defined by the Lawshe model,(12) modified by Tristán,(13) that is CVI > 0.58.

**Table 3:** Content validity index by expert

Expert	Global CVI
1	0.525
2	0.76
3	0.85
4	0.645
5	0.83
6	0.675

The parameters *Nutritional decline (Weight loss > 10%), Cognitive decline (Loss ≥ 5 minimental or ≥ 3 Pfeiffer), >2 advanced chronic diseases or conditions (from the list of specific indicators), Assessment of the intervention demand or intensity: > 2 emergency or unplanned admissions (six months), Assessment of the intervention demand or intensity: increased intervention demand or intensity (home care, nursing interventions, etc.)* were not considered acceptable by the experts; however, the instrument’s CVI is 0.68.

Although the parameters *Functional decline (Karnofsky or Barthel deterioration > 30%* and *Functional decline (loss of >2* ADLs*)* and *Karnofsky <50 or Barthel <20* did not reach the cut-off point, the experts coincided in that the Karnofsky and Barthel scales are not validated and adequate clinical tools to apply in children and adolescents with cancer, which is why they suggested in the observation to replace them with the Lansky scale.

Bearing in mind the results obtained, the work eliminated eight items that did not comply with that established by the Lawshe model(12) modified by Tristán;(13) according to expert consensus, who did not consider these items pertinent to identify palliative needs in children and adolescents from 8 to 17 years of age with cancer.

In the final version, the instrument was integrated with the SQ *Would you be surprised if this patient died within the next year* and eight parameters belonging to four dimensions distributed, thus: the dimension *Demand or Need* was comprised by the parameters *Demand: Has there been any implicit expression or limitation of therapeutic effort or demand for palliative care from the patient, family, or staff members?* and *Need: identified by the professional staff members*, the dimension *Persistent symptoms* remained with the parameter *Symptom checklist (ESAS) ≥ 2 persistent or refractory symptoms*, the dimension *Psychosocial aspects* was integrated by both parameters from the original version *Emotional Discomfort Detection (EDD) > 9* and *Family and social assessment* and the dimension *Specific disease severity/progression indicators* was comprised by the parameters *Oncological disease (Advanced locoregional or metastatic cancer)*, *Oncological disease (in progression (in solid tumors)* and *Oncological disease (Poorly controlled or refractory persistent symptoms despite optimizing specific treatment)*. The final results are indicated in Table 4.

**Table 4:** NECPAL CCOMS ICO instrument for pediatric and adolescent population

*Surprise question (SQ)*	*Would you be surprised if this patient died within the next year?*	*Would you be surprised if this patient died within the next year?*
Dimension	Parameter	Parameter
Demand” or “Need”	Demand: Has there been any implicit expression or limitation of therapeutic effort or demand for palliative care from the patient, family, or staff members?	Demand: Has there been any implicit expression or limitation of therapeutic effort or demand for palliative care from the patient, family, or staff members?
Demand” or “Need”	Need: Identified by professional staff members	Need: Identified by professional staff members
Persistent symptoms	Pain, weakness, anorexia, dyspnea, digestive	Symptoms checklist (ESAS) ≥ 2 persistent or refractory symptoms
Psychosocial aspects	Distress and/or Severe adjustment disorder	Emotional Discomfort Detection (EDD) > 9
Psychosocial aspects	Severe social vulnerability	Family and social assessment
Specific disease severity/progression indicators	Oncological disease	Advanced locoregional or metastatic cancer
Specific disease severity/progression indicators	Oncological disease	Cancer in progression (in solid tumors)
Specific disease severity/progression indicators	Oncological disease	Poorly controlled or refractory persistent symptoms despite optimizing specific treatment

## Discussion

The Lawshe model,(12) modified by Tristán(13) to conduct the content validity process of an instrument, has become useful in this study to determine expert consensus; this methodology has been used successfully by other authors, like Vesga and Ruiz,(20) who evaluated the validity and reliability of a professional care scale in Spanish, finding that the tool obtained a CVI of 0.893 and validity for each of the parameters that exceeds the values established in the literature. Likewise, in 2019 Castro A(21) validated the Spanish version of the instrument Jefferson Scale of Attitudes toward Physician - Nurse Collaboration (JSAPNC) by using the Lawshe model modified by Tristán, obtaining a CVI of 0.84.

Following the same methodology, Cruz and Muñoz,(22) validated an instrument to identify the level of vulnerability of health workers to tuberculosis in health institutions (IVTS TB-001) obtaining a CVI of 0.91. Moreover, Corredor Parra,(23) presents a study titled *Validity and reliability of the instrument of quality of life by Betty Ferrell, for people with chronic disease*, which was a study of six dimensions, with 41 items with CVR with high values ranging between N = (0.77 - 1); only 14 items did not have an adequate value to include them in the results, given that they were a value ranging between N = (0.11 - 0.55). The statistical test used was the CVI by Lawshe modified by Tristán- López, it was determined an adequate content validity with CVI of N = (0.9), an adequate CVI to use in the local context, which demonstrates that the CVI by Lawshe modified by Tristán- López, is widely used.

The surprise question *Would you be surprised if this patient died within the next year* and the parameter *Need: Identified by professional staff members*, validated as pertinent to identify palliative needs in Colombian children and adolescents with cancer, coincide with affirmations by other authors,(11) upon considering terminality as a factor that undoubtedly proposes the need for palliative care or end-of-life care. Similarly, the parameter *Demand: Has there been any implicit expression or limitation of therapeutic effort or demand for palliative care from the patient, family, or staff members* has been documented by other authors who highlight even the importance of the palliation as purpose of any useful therapeutic intervention.(24) In this respect, the Spanish Society of Outpatient Pediatrics and Primary Care (25) has emphasized on the importance of consensus with the family on the importance of not continuing futile invasive maneuvers that temporarily prolong life at the expense of patient suffering.

Depending on the child’s age, the degree of compromise of the disease, the dynamic status and capacity to carry out activities both personally and with the people around them, the Lansky scale is evaluated. This scale is measured through scores, every 10 points correspond to a different category that ranges from 10 to 100, with 10 being the most-severe restriction score and 100, which suggests optimal conditions in their functional state, given that in them it can be difficult to apply the criteria of the Karnofsky and Eastern Cooperative Oncology Group (ECOG) scales that measure the quality of life in oncology patients.(26) Hence, the Lansky scale provides quantifiable, reproducible, and significant data, necessary for effective monitoring and management of children with cancer, having proven even useful to demonstrate significant improvement in functionality and independence after months of chemotherapy in minors with lymphomas and miscellaneous tumors compared with others suffering leukemia, tumors of the central nervous system, and other solid tumors.(27)

In turn, the persistence and refractoriness of symptoms in a child with cancer, identified in the parameter *Pain, weakness, anorexia, dyspnea, digestive… Symptom checklist (ESAS) >2 persistent or refractory symptoms,* was found valid by the experts participating in the study as a component of the 2017 NECPAL CCOMS-ICO© 3.1 instrument that identifies palliative needs in this population group. The aforementioned coincides with that reported by distinct authors(28) who highlight that not only the diagnosis or prognosis can increase this need along with the treatment, mentioning the high prevalence of nausea, vomit, and pain in the pediatric patient with chemotherapy and radiotherapy, with palliative sedation standing out as alternative to control hard-to manage symptoms, especially in end-of-life scenarios. (29)

The parameters *Distress and/or Severe adjustment disorder; Emotional Discomfort Detection (EDD);* and *Severe social vulnerability; Family and social assessment* were accepted by the experts because socio-family vulnerability and emotional discomfort are determining factors during the course of the disease. This agrees with that manifested by other researchers (30,31) who have documented the emotional experience in pediatric patients as a burden that sometimes includes unpleasant sensations, like discomfort, sleep problems, crying, and apprehension to the treatment, adding to the above the change in social and family roles, where the parents perceive more negative interactions strongly associated with stress, given that they refer to greater work conflicts when missing work due to their children’s disease.

The parameters *Oncological disease: Advanced locoregional or metastatic cancer, Oncological disease: in progression (in solid tumors)* and *Oncological disease: Poorly controlled or refractory persistent symptoms despite optimizing specific treatment* were found pertinent by the experts to identify palliative needs in Colombian children and adolescents from 9 to 17 years of age with cancer. In this regard, other authors have pointed to neoplasia as the second cause of death in children > 1 year of age, highlighting that solid tumors (lung carcinoma, osteosarcoma, thyroid carcinoma, rhabdomyosarcoma, teratocarcinoma, melanoma, and Wilms tumor) are associated with higher mortality compared with leukemia, besides the increased risk of metastasis and poor prognosis according to stage. (32)

Furthermore, the findings herein coincide with some of the items and domains of the PaPas Scale,(11) an instrument validated in English to identify palliative needs in pediatric population, given that item 1.2 from this instrument corresponds to domain 1 *Life expectancy* also tries to establish the prognosis perceived by the treating professional, as well as the surprise question from the NECPAL CCOMS-ICO© version 3.1 2017. Items 4.1 and 4.2 from domain 4 *Burden of symptoms and problems* of the PaPaS Scale, on the number and intensity of symptoms, agree with the parameter *Symptom checklist (ESAS) ≥ 2 persistent or refractory symptoms,* as well as items 4.3, 4.4 and 4.5 *Psychological distress of the patient, Psychological distress of the parents and Psychological distress of the siblings* from domain 4 *Burden of symptoms and problems* of the PaPas Scale that resemble the parameters *Distress and/or Severe adjustment disorder: Emotional Discomfort Detection (EDD) > 9* and *Severe social vulnerability: Family and social assessment*, all considered valid by the experts participating in the assessment process.

Consequently, palliative care is appropriate for children and adolescents suffering from a highly life-threatening disease, like cancer, where - according to its stage - there are unpleasant symptoms, like pain and other symptoms, which require not only medical support, but also social, spiritual, and psychological attention during the illness and bereavement to improve the quality of life of minors and their families. The need to have instruments to identify palliative needs is fundamental to establish criteria to identify these palliative needs in different environments, from the early phases of the disease to the end of life, favoring an early and quality palliative approach upon a scenario of oncological disease in the pediatric and adolescent population.

Finally, the authors in this study recognize as limitation that the number of pediatric palliative care in Colombia is scarce, which represents difficulties when wishing to have a considerable number of experts to conduct the content validity process, thus highlighting that - to date - Colombia has no formation programs in pediatric palliative care for professionals in health sciences.

In conclusion, the 2017 NECPAL CCOMS-ICO© version 3.1 instrument’s content validity in Spanish proved appropriate to identify palliative needs in Colombian children and adolescents from 8 to 17 years of age with cancer with a CVI of 0.68. The study provides a valid instrument from the consensus by experts to identify palliative needs in the study’s target population for the Colombian context, validated in Spanish. These results enrich the nursing discipline by generating knowledge in the field of pediatric palliative care and, specifically, in validation processes of instruments, recognizing that the 2017 NECPAL CCOMS-ICO© version 3.1 questionnaire for Colombian children and adolescents could be used by distinct specialties to identify palliative needs in Colombian children and adolescents with cancer from 8 to 17 years of age.
